# Comparison of Outcomes after Phacoemulsification with Two Different Corneal Incision Distances Anterior to the Limbus

**DOI:** 10.1155/2019/1760742

**Published:** 2019-08-19

**Authors:** Lijun Wang, Lin Zhao, Xiting Yang, Yi Zhang, Dingying Liao, Jianming Wang

**Affiliations:** Department of Ophthalmology, The Second Affiliated Hospital of Xi'an Jiaotong University, 157 Xiwu Road, Xi'an 710004, China

## Abstract

**Purpose:**

To compare visual performance and visual quality outcomes after phacoemulsification with two different clear corneal incision (CCI) distances anterior to the limbus in senile cataract patients.

**Methods:**

Retrospective case series. Patients who had undergone phacoemulsification were divided into two groups according to the CCI distances anterior to the limbus. The CCI distances in group A range from 1 mm to 1.5 mm, while those in group B range from 0.5 mm to 1 mm. The visual acuity, refraction, surgically induced astigmatism (SIA), corneal aberrations, anterior segment parameters, and subjective vision quality were evaluated.

**Results:**

This study enrolled 54 eyes, with 27 eyes per group. Both groups had significant improvement in postoperative uncorrected visual acuity (UCVA) and corrected distance visual acuity (CDVA) (*P* < 0.05). There were no statistically significant between-group differences in postoperative UDVA, CDVA, SIA, corneal aberrations, anterior segment parameters, or VF-QOL questionnaire performance (*P* > 0.05).

**Conclusions:**

The phacoemulsification with CCI distances ranging from 0.5 mm to 1.5 mm is an effective and safe therapy to senile cataract. The CCI distance anterior to the limbus that ranges from 0.5 mm to 1.5 mm is recommended for routine phacoemulsification.

## 1. Introduction

Today, the technology of cataract treatment has been further advanced. The clear corneal incision (CCI) phacoemulsification has become a mainstream treatment for cataract worldwide. CCI has attracted the attention of researchers widely, for it plays a key role in cataract surgery process and postoperative visual performance. Within the past few years, many trials of CCI in phacoemulsification have studied the effect of size and axis of CCI. These studies have proved that CCI sizes and axes have significant effects on astigmatism and visual function after phacoemulsification [1–3]. However, it is difficult to say presently that whether the CCI distance anterior to the limbus affects the outcomes after phacoemulsification or not. There is a lack of recommendation or uniform rule on the CCI distance anterior to the limbus. The CCI distance anterior to the limbus described in the literature varies from each other, such as 0.5 mm [4] and 1 mm [5]. Then, some scholars choose the position of the edge of the limbal vessels to process CCI [6, 7], but the position of the edge of the limbal vessels of different patients often differs, which results in a variable of CCI distance. Moreover, CCI distance of different patients after phacoemulsification is different from person to person in clinical. It is obvious that the further the CCI is anterior to the limbus, the closer it is to the visual axis. However, the safe distance range of CCI is questionable. Hence, the purpose of this study is to evaluate the effect of two different CCI distances on phacoemulsification by comparing visual acuity, surgically induced astigmatism (SIA), corneal aberrations, anterior segment parameters, and subjective vision quality.

## 2. Methods

### 2.1. Study Design

This retrospective case series was performed at the Department of Ophthalmology, the Second Affiliated Hospital of Xi'an Jiaotong University, Xi'an, China. Clinical data were collected retrospectively from patients who had undergone phacoemulsification and IOL implantation between February 2017 and April 2018. This study was approved by the hospital's ethics committee (2017, no. 004) and was performed in accordance with the tenets of the Declaration of Helsinki.

### 2.2. Inclusion and Exclusion Criteria

Patients with senile cataract, older than 50 years, underwent phacoemulsification and IOL implantation were included. The exclusion criteria were corneal astigmatism greater than 2.00 diopters (D), corneal disorders, pterygium, glaucoma, uveitis, retinal disorders, congenital eye abnormality, a history of eye trauma, previous ocular surgery, and intraoperative complications.

### 2.3. Case Grouping

Three months after surgery, the slit-lamp microscopic examination was performed with the patient in a sitting position to determine the CCI distance anterior to the limbus. Eyes with 1 mm to 1.5 mm incision distances were assigned to group A, and eyes with 0.5 mm to 1 mm incision distances were assigned to group B.

### 2.4. Surgical Technique

Informed consent was obtained from every patient before surgery. All operations were performed by experienced surgeons using Infiniti phacoemulsifier. After the application of topical anesthesia (proparacaine hydrochloride 0.5%), a self-sealing 3.0 mm clear corneal incision was made at 10∼11 o'clock, and a side-port incision was made at 2 o'clock. 5.5 mm diameter continuous curvilinear capsulorhexis (CCC) was made, hydrodelineation and hydrodissection were performed, and routine phacoemulsification was performed, after which a foldable IOL was implanted in the capsular bag. The incision was hydrated to aid the incision closure. No eye required sutures. All patients received routine postoperative topical steroids (TobraDex, Alcon, Belgium) and antibiotic eye drops (Levofloxacin Eye Drops, Santen, Japan) for 4 weeks.

### 2.5. Preoperative and Postoperative Examinations

A complete ophthalmology examination was performed on each patient preoperatively, including visual acuity, slit-lamp microscope, intraocular pressure (CT-1P, Topcon, Tokyo, Japan), phoropter (KR-8900, Topcon, Tokyo, Japan), A/B-scan ultrasonography (SW-2100, SUOER, Tianjin, China), specular microscope (SP-01, C.S.O.SRL, Italy), visual electrophysiology (Scan 21, Roland Consult Stasche & Finger GmbH, Brandenburg, Deutschland), and dilated funduscopic examination. Preoperative IOL calculations were performed on the basis of IOL Master (Carl Zeiss Meditec AG, Jena, Germany) and A-scan ultrasonography measurements. The corneal topography (Pentacam, Oculus, Wetzlar, Germany) and optical coherence tomography (OCT) (Cirrus HD-OCT, Carl Zeiss Meditec Inc., California, USA) examinations were performed before surgery as well as 3 months after surgery.

### 2.6. Main Outcome Measures

The CCI distance was measured by the caliper at 3 months postoperatively. To minimize interobserver variability, a single operator (J.W.) acquired all distances. The uncorrected visual acuity (UCVA) and corrected distance visual acuity (CDVA) performed by an optometrist were recorded. Then, decimal visual acuity was converted to logMAR scale for statistical analysis. The refractive spherical and cylindrical power and spherical equivalent (SE) were examined using the KR-8900 device. Corneal astigmatism was assessed by the Pentacam. The corneal SIA was calculated by vector analysis on the basis of the results of corneal curvature (K1 and K2) and axis measured by the Pentacam. The preoperative and postoperative anterior chamber depth (ACD), anterior chamber angle (ACA), and central corneal thickness (CCT) were measured using the Pentacam. The preoperative and postoperative root mean square (RMS) of higher-order aberration (HOA), spherical aberration (*Z*_4_^0^), oblique trefoil (*Z*_3_^3^), vertical trefoil (*Z*_3_^−3^), horizontal coma (*Z*_3_^1^), and vertical coma (*Z*_3_^−1^) measured by the Pentacam were recorded. Postoperative complications were recorded. Subjective vision quality was evaluated with the vision function (VF) and quality of life (QOL) questionnaires. Patients completed the self-administered questionnaire at 3 months postoperatively. Generally, questionnaires were completed by the patients without assistance. However, if the patients requested, explanations of the questions were given. The VF-QOL questionnaire contains 25 questions, assessing nine subscales (general, visual perception, peripheral vision, sensory adaptation, depth perception, self-care, mobility, social, and mental). For each question, the 4-point rating scale was scored from 1 (no problems) through 4 (maximum problems), with 2 and 3 for the intermediate rankings. Then, scales were converted into a 100-point scale for statistical analysis, with 100 being the best score and 0 the worst score [8].

### 2.7. Statistical Analysis

Statistical analysis was performed using SPSS software (version 19 for Windows, SPSS Inc.). Differences in sex and eye between groups were analyzed using the chi-square test. The *t* test was used to analyze the age, astigmatism, SIA, visual acuity, refraction, ACD, ACA, CCT, and aberration between groups. The paired *t* test was used to make an analysis of preoperative data and postoperative data. A *P* value less than 0.05 was considered statistically significant.

## 3. Results

### 3.1. Demographics

This study enrolled 54 eyes of 38 patients, with 27 eyes per group.  Table 1 shows the patients' demographics and preoperative characteristics. The mean age of patients was 63.59 ± 8.82 years in group A and 68.04 ± 9.61 years in group B. The sex ratio (male/female) was 16/11 in two groups. There were no statistically significant differences between the two groups in age, sex ratio, eyes, UCVA, IOP, or astigmatism (*P* > 0.05).

### 3.2. Visual Acuity and Refraction Outcomes

In both groups, there were significant improvements in the UCVA at 1 day postoperatively (0.16 ± 0.17 logMAR and 0.20 ± 0.13 logMAR in the A and B groups, respectively) (*P* < 0.05) (Table 2) and 3 months postoperatively (0.16 ± 0.16 logMAR and 0.19 ± 0.15 logMAR in the A and B groups, respectively) (*P* < 0.05) (Table 2). The CDVA significantly improved at 3 months postoperatively (0.03 ± 0.07 logMAR and 0.06 ± 0.09 logMAR in the A and B groups, respectively) (*P* < 0.05) (Table 2). However, no statistically significant differences were detected between the two groups in the postoperative UCVA, CDVA, mean UCVA increase, or mean CDVA increase (*P* > 0.05) (Table 2). There were no statistically significant differences between the two groups in the sphere, subjective cylinder, or SE (*P* > 0.05) (Table 2).

### 3.3. Corneal Astigmatism

The preoperative and postoperative corneal astigmatism measurements are shown in Table 3. There was no significant change in corneal astigmatism from preoperatively to postoperatively (*P* > 0.05). Differences in corneal astigmatism between groups were not significant at any corneal zone (4.0 mm, 6.0 mm, or 8.0 mm) (*P* > 0.05).

### 3.4. SIA

**Figure 1:**
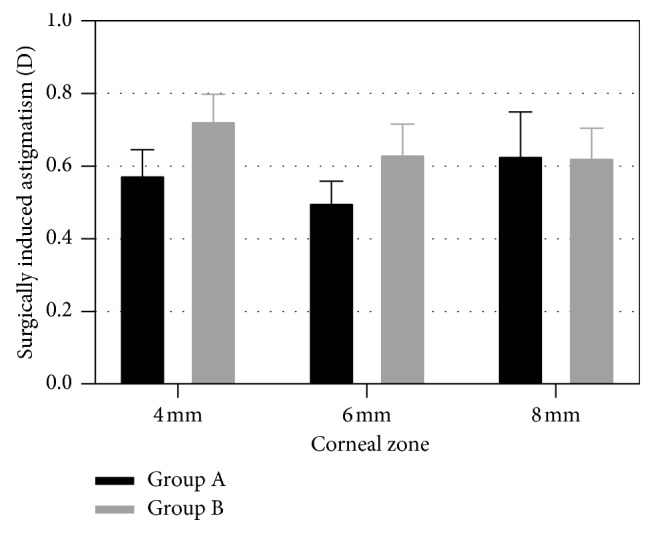
Surgically induced astigmatism in the two groups.

Figure 1 shows the comparison of SIA between groups in different corneal zones. No statistically significant differences were found between the two groups in the 4 mm central corneal SIA (0.58 ± 0.34 D and 0.73 ± 0.35 D in the A and B groups, respectively) (*P*=0.13), 6 mm central corneal SIA (0.50 ± 0.34 D and 0.63 ± 0.40 D in the A and B groups, respectively) (*P*=0.22), or 8 mm central corneal SIA (0.63 ± 0.59 D and 0.63 ± 0.40 D in the A and B groups, respectively) (*P*=0.97).

### 3.5. Aberrations

**Figure 2:**
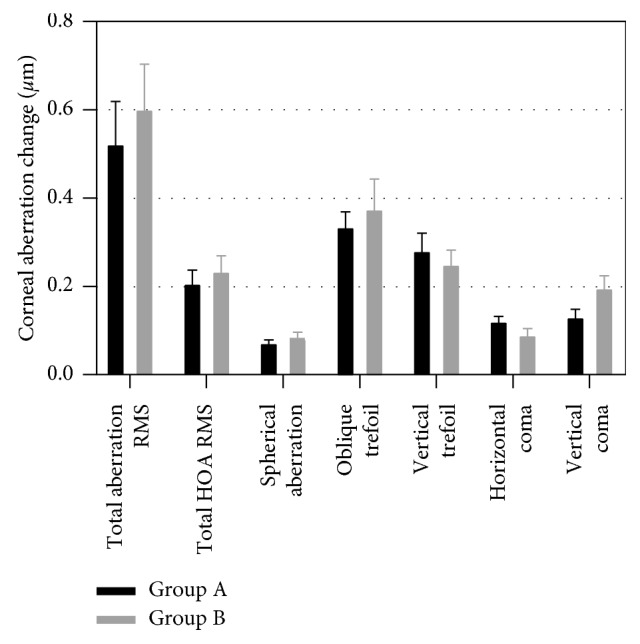
Corneal aberration changes in the two groups.

Table 4 shows the patients' corneal aberrations, and Figure 2 shows the corneal aberration changes from preoperatively to postoperatively in the two groups. In group A, there were significant changes in the total HOA RMS and vertical trefoil (*Z*_3_^−3^) from preoperatively to postoperatively (*P* < 0.05) (Table 4). In group B, there were significant changes in the total aberration RMS and horizontal coma (*Z*_3_^1^) from preoperatively to postoperatively (*P* < 0.05) (Table 4). However, no statistically significant differences of aberration changes from preoperatively to postoperatively between the two groups were found in the total aberration RMS, total HOA RMS, spherical aberration (*Z*_4_^0^), oblique trefoil (*Z*_3_^3^), vertical trefoil (*Z*_3_^−3^), horizontal coma (*Z*_3_^1^), and vertical coma (*Z*_3_^−1^) (*P* > 0.05) (Figure 2).

### 3.6. Anterior Segment Parameters

In both groups, compared with the preoperative measurements, the postoperative ACD was statistically significantly deeper (*P* < 0.001) (Table 5), and the postoperative ACA increased significantly (*P* < 0.001) (Table 5). Then, there was a significant change in the CCT from preoperatively to postoperatively (*P* < 0.01) (Table 5). However, no statistically significant between-group differences were found in the ACD, AVA, or CCT at preoperatively or postoperatively (*P* > 0.05) (Table 5).

### 3.7. Vision Function and Quality of Life

**Figure 3:**
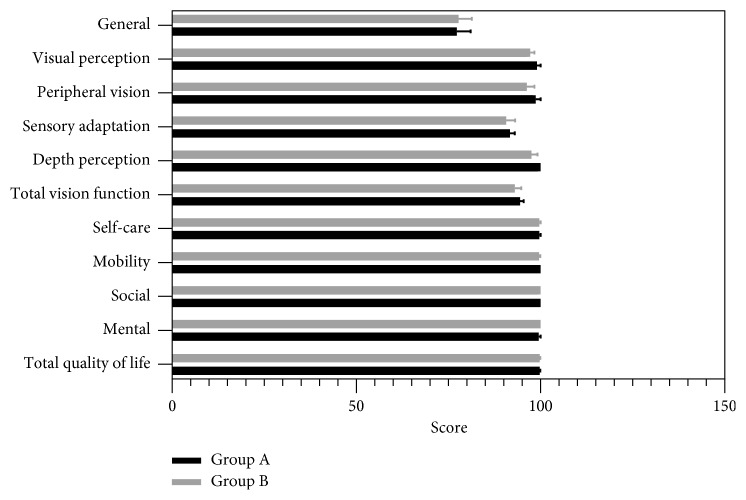
Results of the VF-QOL questionnaire in the two groups (high values indicate favorable result).

Figure 3 shows a comparison of VF-QOL scores between the two groups. Group A had a slightly higher mean score on the VF questionnaire (94.42 ± 5.08 and 93.04 ± 9.12 in the A and B groups, respectively). Then, group B had a slightly higher mean score on the QOL questionnaire (99.78 ± 0.80 and 99.79 ± 0.74 in the A and B groups, respectively). However, these differences were not statistically significant (*P*=0.51 and *P*=0.94, respectively). No statistically significant differences between the two groups were found in the general, visual perception, peripheral vision, sensory adaptation, depth perception, self-care, mobility, social, or mental subscale (*P* > 0.05) (Figure 3).

### 3.8. Complications

No eye had persistent corneal edema, significant intraocular pressure increase, IOL displacement, clinically significant macular edema, retinal detachment, endophthalmitis, intraocular hemorrhage, or other severe postoperative complications.

## 4. Discussion

In this study, we assessed the results of phacoemulsification with two different CCI distances in eyes with senile cataract and analyzed SIA, corneal aberrations, anterior segment parameters, and subjective vision quality in groups. We wish to see whether different CCI distances anterior to the limbus have influences on cataract surgery efficacy.

Cornea is an important optical refractive medium. CCI is generally known to induce the cornea changes in shape and biomechanics. Then, CCI influences the corneal astigmatism and aberrations [1]. In previous studies [1–3], different sizes and axes (temporal, superotemporal superior, and on-axis) of corneal incisions could induce significant differences in the corneal structure and refraction. In our study, we compared the effect of two different CCI distances on phacoemulsification; however, no significant differences were detected.

The goal of modern cataract surgery is to achieve fast visual rehabilitation without complications and with low postoperative residual refractive errors [9]. We assessed the visual acuity and refraction by analyzing UCVA, CDVA, sphere, cylinder, and spherical equivalent. Our results showed that both phacoemulsification incisions performed well in terms of visual and refractive outcomes. The postoperative UCVA, CDVA, and SE were the same in both groups.

Corneal incision could induce coupled flattening and steepening effect on corneal curvature, flattening of the meridian of the incision, and steepening along the orthogonal meridian [10, 11]. Changes in corneal curvature lead to corneal astigmatism changes. The SIA is an objective and comprehensive outcome to assess the astigmatism change resulted from surgery. Thus, we used SIA to evaluate the degree of CCIs' influence on corneal astigmatism. The SIA was calculated on the basis of the results of preoperative and postoperative corneal curvature and axis. Previous studies have proved that corneal incision size, incision axis, and incision location all have influence on SIA [1, 2, 12]. Theoretically, change in CCI distance has a significant influence on SIA, for the curvature of the peripheral cornea is flatter than the center cornea, and the closer the corneal incision is to the visual axis, the greater the effect is on SIA [12]. However, in our study, no significant differences were found between the two distance CCI groups in SIA of different corneal zones, indicating that the two CCI distances have a similar impact on corneal astigmatism.

Wavefront analysis is an objective measurement of visual quality. The decline of visual quality is related to the aberrations [13]. Aberrations can be divided into low-order aberrations (sphere and cylinder) and high-order aberrations. Approximately 93% of the aberration in a normal eye is known to be attributable to the lower-order aberrations which limit the visual ability of eyes [14]. However, HOAs are also important in achieving the best optical quality in pseudophakic eyes [14, 15]. We compared the wavefront parameters of 3rd-order trefoil, coma, and 4th-order spherical aberrations, for these aberrations constitute the major component of HOAs, and HOAs greater than the 6th Zernike polynomial do not make a significant clinical contribution [14–17]. Furthermore, wavefront analysis might predict visual complaints. For example, glare was found to be associated with higher total HOAs and spherical aberration [18]. Spherical aberration is an important factor affecting retinal imaging and is related to contrast sensitivity [19]. Corneal coma stands for the refractive difference between both sides of the cornea [15]. In our study, both phacoemulsifications with different incisions caused an increase in postoperative high-order aberrations, which are consistent with previous research results [10, 20]. These outcomes result from corneal incision increasing the irregularity of corneal shape. However, the degree of the change in each high-order aberration is similar in the two distance CCI groups.

We assessed the changes in the anterior segment by analyzing the ACD, ACA, and CCT measured by the Pentacam. Cataract surgery can cause changes in the anterior segment of the eye [21, 22]. Previous studies have shown that postoperative refraction and refractive stability are closely related to the ACD, ACA, axial length, and so on [21, 23]. Olsen has reported that ACD is a crucial factor affecting postoperative refraction [24]. A change of 1 mm in postoperative ACD could produce a change of 1.5 D in refraction [24]. Obviously, phacoemulsification and IOL implantation could deepen the ACD and widen the ACA, for the IOL is a well-known thinner than the lens of the human. Our results are consistent with those; however, the changes in ACD and ACA between the two distance CCI groups have no significant differences. We evaluated the changes in CCT to determine the effect of the techniques on cornea tissue. No patient suffered corneal edema according to the results of CCT. Actually, the CCT decreases at 3 months after surgery significantly, and then, the two distance CCI groups have comparable results.

Subjective vision quality is also an essential component to assess the visual performance. Some patients with significant improvement of clinical vision after surgery are dissatisfied with the outcomes because their expectations were not met or their postoperative visual quality is limited. Glare and halo, common optical side effects after surgery, can significantly affect patient's visual performance and patient's satisfaction [25]. For this reason, the assessment of subjective vision quality is necessary and could be used as a complementary tool to confirm a successful outcome after cataract surgery. In this study, we assessed the subjective vision quality by using the VF-QOL questionnaire, which is a validated questionnaire and suitable for the developing countries [8, 26, 27]. The results showed that patients in two distance CCI groups have similar experience on visual perception, peripheral vision, sensory adaptation, depth perception, self-care ability, mobility ability, social function, and mental function.

Our study had limitations. First, we were not clear about all the details of the surgery process (such as phacoemulsification time) for its retrospective nature. Second, we did not assess contrast sensitivity and corneal endothelial cell, which are also important to evaluate the efficacy of phacoemulsification. Third, the effect of CCI distance exceeding 1.5 mm on phacoemulsification needs further research. Additional research is needed to confirm the current findings. Fourth, the limited number of patients enrolled would reduce the statistical power of the analysis. Then, long-term outcomes are necessary.

## 5. Conclusion

In conclusion, whether CCI distance ranges from 1 mm to 1.5 mm or from 0.5 mm to 1 mm, the phacoemulsification is an effective and safe therapy on cataract. Both techniques can significantly improve the visual performance with fewer complications. Hence, we recommend a CCI distance of 0.5 mm to 1.5 mm anterior to the limbus for routine phacoemulsification.

## Figures and Tables

**Table 1:** Demographics and preoperative data in the two groups (mean ± SD).

Group	Case	Age (years)	Sex (M/F)	Eye (R/L)	UCVA (logMAR)	IOP (mmHg)	Astigmatism (D)
Group A	27	63.59 ± 8.82	16/11	14/13	0.80 ± 0.48	15.24 ± 4.07	0.87 ± 0.47
Group B	27	68.04 ± 9.61	16/11	14/13	0.94 ± 0.64	15.79 ± 3.28	0.87 ± 0.51
*P* value		0.08	1.00	1.00	0.38	0.58	0.96

UCVA = uncorrected visual acuity.

**Table 2:** Visual acuity and refractive outcomes in the two groups (mean ± SD).

Parameter	Group A	Group B	*P* value
Preoperative UCVA	0.80 ± 0.48	0.94 ± 0.64	0.38
Postoperative UCVA (1 d)	0.16 ± 0.17^*∗*^	0.20 ± 0.13^*∗*^	0.31
Postoperative UCVA (3 mo)	0.16 ± 0.16^*∗*^	0.19 ± 0.15^*∗*^	0.54
Postoperative CDVA (3 mo)	0.03 ± 0.07^*∗*^	0.06 ± 0.09^*∗*^	0.26
Mean UCVA increase	−0.64 ± 0.49	−0.75 ± 0.68	0.49
Mean CDVA increase	−0.77 ± 0.50	−0.89 ± 0.64	0.48
Sphere (D)	0.23 ± 1.33	0.25 ± 0.88	0.93
Subjective cylinder (D)	−1.40 ± 0.83	−1.51 ± 1.07	0.68
SE (D)	−0.47 ± 1.21	−0.50 ± 0.60	0.92

UCVA = uncorrected visual acuity; CDVA = corrected distance visual acuity; SE = spherical equivalent. ^*∗*^Values are statistically significant compared with preoperative examinations (*P* < 0.05).

**Table 3:** Corneal astigmatism in the two groups (mean ± SD).

Corneal astigmatism zone	Preoperative	Postoperative	*P* value
4.0 mm			
Group A	0.86 ± 0.54	0.92 ± 0.70	0.57
Group B	0.86 ± 0.56	0.94 ± 0.62	0.43
*P* value	0.98	0.92	

6.0 mm			
Group A	0.81 ± 0.42	0.88 ± 0.61	0.41
Group B	0.87 ± 0.55	0.90 ± 0.50	0.67
*P* value	0.69	0.90	

8.0 mm			
Group A	0.84 ± 0.48	1.02 ± 0.74	0.16
Group B	0.90 ± 0.52	0.84 ± 0.52	0.46
*P* value	0.65	0.35	

**Table 4:** Corneal aberrations in the two groups (*μ*m, mean ± SD).

	Preoperative	Postoperative (3 mo)	*P* value
Total aberration RMS			
Group A	2.760 ± 1.624	2.753 ± 1.758	0.96
Group B	2.825 ± 2.040	3.140 ± 2.063	0.04^*∗*^
*P* value	0.90	0.48	

Total HOA RMS			
Group A	0.875 ± 0.383	1.014 ± 0.402	0.006^*∗*^
Group B	0.883 ± 0.476	0.976 ± 0.428	0.13
*P* value	0.95	0.75	

Spherical aberration (*Z*_4_^0^)			
Group A	0.442 ± 0.193	0.423 ± 0.197	0.274
Group B	0.408 ± 0.217	0.395 ± 0.189	0.54
*P* value	0.56	0.61	

Oblique trefoil (*Z*_3_^3^)			
Group A	−0.014 ± 0.193	−0.149 ± 0.359	0.08
Group B	0.020 ± 0.246	0.003 ± 0.401	0.87
*P* value	0.59	0.16	

*Vertical trefoil* (*Z*_3_^−3^)			
Group A	−0.199 ± 0.302	−0.394 ± 0.274	0.003^*∗*^
Group B	−0.171 ± 0.241	0.259 ± 0.351	0.15
*P* value	0.71	0.13	

Horizontal coma (*Z*_3_^1^)			
Group A	−0.054 ± 0.195	−0.041 ± 0.206	0.66
Group B	0.017 ± 0.183	0.075 ± 0.180	0.016^*∗*^
*P* value	0.19	0.04^*∗*^	

Vertical coma (*Z*_3_^−1^)			
Group A	0.345 ± 0.332	0.312 ± 0.315	0.33
Group B	0.331 ± 0.535	0.303 ± 0.463	0.59
*P* value	0.91	0.94	

RMS = root mean square; HOA = higher-order aberration. ^*∗*^Statistically significant at *P* < 0.05.

**Table 5:** Anterior segment parameters in the two groups (mean ± SD).

Parameter	Preoperative	Postoperative	*P* value
ACD (mm)			
Group A	2.72 ± 0.46	4.08 ± 0.47	<0.001^*∗*^
Group B	2.88 ± 0.46	3.85 ± 0.86	<0.001^*∗*^
*P* value	0.24	0.25	

ACA (°)			
Group A	35.35 ± 10.67	44.21 ± 4.56	<0.001^*∗*^
Group B	34.68 ± 8.22	43.30 ± 5.08	<0.001^*∗*^
*P* value	0.81	0.51	

CCT (*μ*m)			
Group A	553.52 ± 27.97	545.92 ± 28.66	0.006^*∗*^
Group B	538.56 ± 36.27	529.48 ± 34.96	0.002^*∗*^
*P* value	0.11	0.08	

ACD = anterior chamber depth; ACA = anterior chamber angle; CCT = central corneal thickness. ^*∗*^Statistically significant at *P* < 0.05.

## Data Availability

The datasets used to support the findings of this study are available from the corresponding author upon request.
